# RETRACTION: Effect of right internal mammary artery versus radial artery as a second graft vessel in coronary artery bypass grafting on postoperative wound infection in patients: A meta‐analysis

**DOI:** 10.1111/iwj.70110

**Published:** 2024-10-16

## Abstract

Retraction: DuH. , GuX. , ZhangZ. , DongZ. , RanX. , and ZhouL. , “Effect of Right Internal Mammary Artery Versus Radial Artery as a Second Graft Vessel in Coronary Artery Bypass Grafting on Postoperative Wound Infection in Patients: A Meta‐Analysis,” International Wound Journal 21, no. 3 (2024): e14592, 10.1111/iwj.14592.38424286 PMC10904365

The above article, published online on 29 February 2024, in Wiley Online Library (http://onlinelibrary.wiley.com/), has been retracted by agreement between the journal Editor in Chief, Professor Keith Harding; and John Wiley & Sons, Ltd. It came to the publisher's attention from a third party that a number of articles shared concerning similarities in format and structure. Following an investigation by the publisher, the retraction has been agreed on as the peer review and publishing process for this article were found to be manipulated. The authors did not respond to the notice of retraction.

Retraction: H. Du , X. Gu , Z. Zhang , Z. Dong , X. Ran , and L. Zhou , “Effect of Right Internal Mammary Artery Versus Radial Artery as a Second Graft Vessel in Coronary Artery Bypass Grafting on Postoperative Wound Infection in Patients: A Meta‐Analysis,” International Wound Journal 21, no. 3 (2024): e14592, 10.1111/iwj.14592.38424286 PMC10904365

The above article, published online on 29 February 2024, in Wiley Online Library (http://onlinelibrary.wiley.com/), has been retracted by agreement between the journal Editor in Chief, Professor Keith Harding; and John Wiley & Sons, Ltd. It came to the publisher's attention from a third party that a number of articles shared concerning similarities in format and structure. Following an investigation by the publisher, the retraction has been agreed on as the peer review and publishing process for this article were found to be manipulated. The authors did not respond to the notice of retraction.

